# Designing and Developing an eHealth Program for Patients With Persistent Physical Symptoms: Usability Study

**DOI:** 10.2196/42572

**Published:** 2023-02-08

**Authors:** Oliver Rønn Christensen, Leonora Hedegaard, Mette Trøllund Rask, Jane Clemensen, Lisbeth Frostholm, Marianne Rosendal

**Affiliations:** 1 Research Clinic for Functional Disorders and Psychosomatics Aarhus University Hospital Aarhus N Denmark; 2 Department of Culture and Learning Aalborg University Aalborg Denmark; 3 Hans Christian Andersen Children’s Hospital Odense University Hospital Odense Denmark; 4 Department of Clinical Medicine Aarhus University Aarhus Denmark; 5 Research Unit for General Practice Aarhus University Aarhus Denmark

**Keywords:** eHealth, digital health, medically unexplained symptom, persistent physical symptom, self-management, usability, physical symptom, persistent symptom, unexplained symptom, symptom management, unguided, thinking aloud, think aloud

## Abstract

**Background:**

Patients with persistent physical symptoms presenting in primary care are often affected by multiple symptoms and reduced functioning. The medical and societal costs of these patients are high, and there is a need for new interventions tailored to both the patients and health care system.

**Objective:**

This study aimed to examine the usability of an unguided, self-help treatment program, “My Symptoms,” developed to assist patients and general practitioners in symptom management.

**Methods:**

In all, 11 users (4 patients with persistent physical symptoms and 7 laypeople) participated in web-based thinking-aloud interviews involving the performance of predefined tasks in the program. Thematic analysis was used to categorize the severity of usability issues. General usability heuristics were cross-referenced with the usability issues.

**Results:**

The analysis identified important usability issues related to functionality, navigation, and content. The study shows how therapeutic knowledge in some cases was lost in the translation of face-to-face therapy to a digital format. The user testing helped uncover how the functionality of the digital elements and general navigation of the program played a huge part in locating and accessing the needed treatment. Examples of redesign to mediate the therapeutic value in the digital format involving health care professionals, web developers, and users are provided. The study also highlights the differences of involving patients and laypeople in the interviews.

**Conclusions:**

Taking the experience of common symptoms as a point of departure, patients and laypeople contributed to finding usability issues on program functionality, navigation, and content to improve the program and make the treatment more accessible to users.

## Introduction

### Background

The experience of physical symptoms is a normal phenomenon. Most symptoms are self-limiting, but they may become persistent and lead to frequent contacts with health care providers. In general practice, 17% of patients are affected by persistent physical symptoms (PPS). These patients have an increased risk of disability and mental comorbidity impacting quality of life, health care use, and the ability to work [1,2].

**Figure 1:**
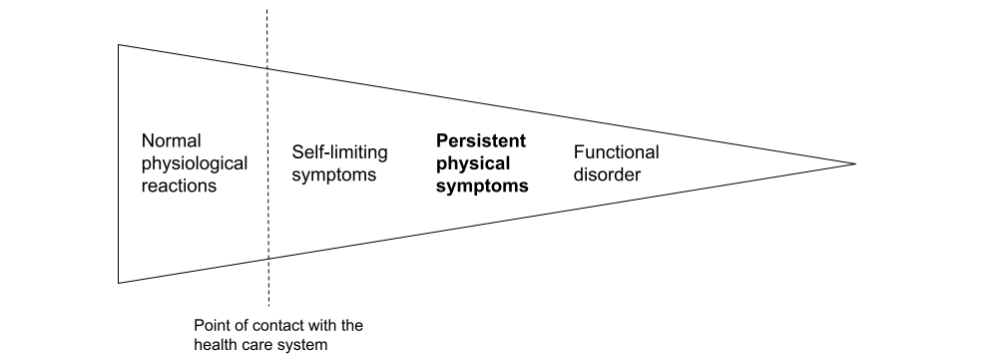
Illness spectrum from symptom to disorders (based on Rosendal et al [7]).

Symptoms span a continuum from mild to severely disabling. This paper refers to symptoms that lead to repeated contact with general practice and may be associated with some degree of functional disability but do not reach the severity of a disease such as a functional disorder (Figure 1). The present Danish national treatment guidelines [3,4] recommend a stepped care model cohering to this continuum in which general practitioners (GPs) are expected to provide care for patients with mild to moderately severe symptoms, that is, PPS. However, specific treatment for PPS in general practice is almost nonexisting [5]. General practice is characterized by high workload and time-restricted consultations, and GPs may tend to focus on investigations to rule out severe disease, without providing guidance to patients on how to manage their symptoms when tests and investigations come out negative [6]. Thus, there is an urgent need to improve the treatment of patients with PPS in primary care to support change in symptom perception and illness behavior to reduce patients’ risk of becoming chronically disabled.

### The My Symptoms Program and Study

**Figure 2:**
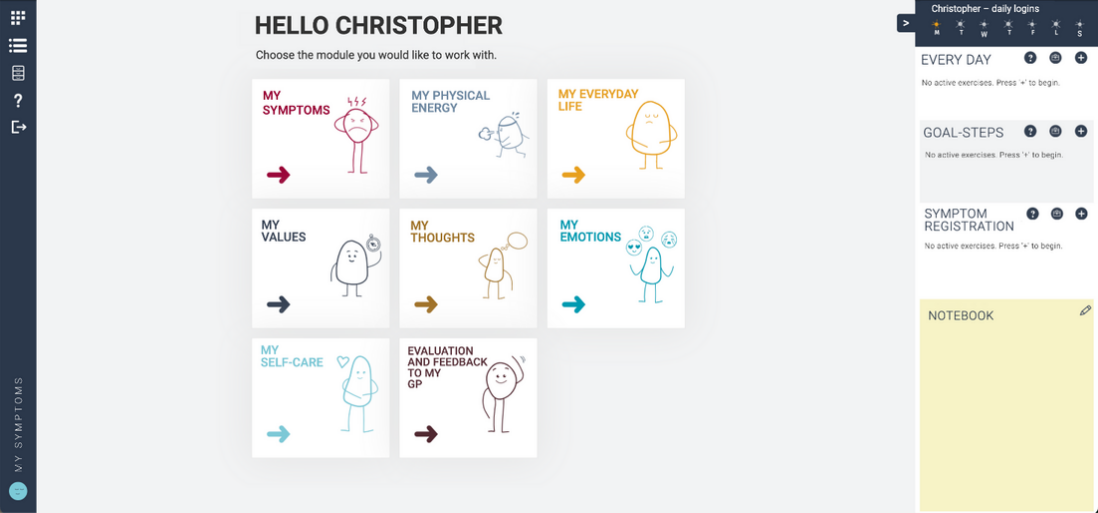
Dashboard of the "My Symptoms" program.

According to previous studies, internet-based self-help interventions may contribute to symptom alleviation and improved quality of life [8,9]. To assist GPs in symptom management and to offer patients with PPS a new treatment option, we developed a novel eHealth program, “My Symptoms.” The program content is inspired by cognitive behavioral therapy. It provides psychoeducation on symptoms and modules on the impact of lifestyle, stress and strain, thoughts, feelings, values, and self-care. Throughout the modules, interactive tools to support behavior change are embedded. The patient can interact with modules on his or her own accord (Figure 2). The content of “My Symptoms” is presented in various forms such as text, pictures, figures, interactive elements, audio, and video. The program is prescribed by the GP but is unguided, that is, no health care professional (HCP) will assist the patient in the use of the program. The program is a responsive web application that is accessible from computers, tablets, and smartphones through a web browser.

**Figure 3:**
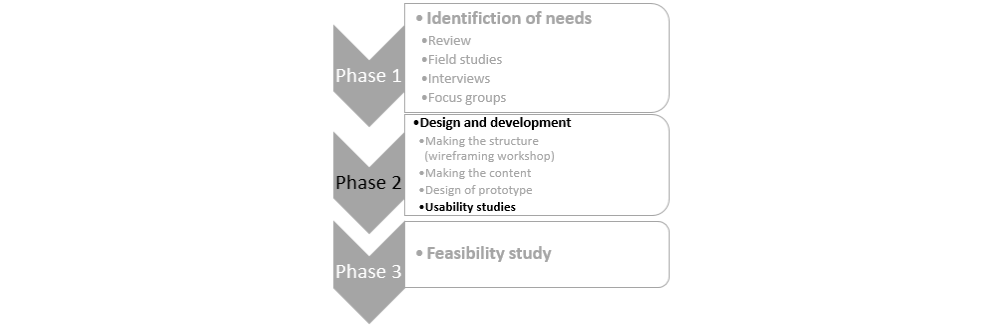
The 3 phases of the participatory design–inspired development of the "My Symptoms" program. Adapted from Jensen et al [12].

The overall framework of making the “My Symptoms” program lent itself to ideas from the participatory design research paradigm within health care [10,11]. Here, emphasis was on the democratization of the development from different stakeholders and participants via iterative processes. The development of “My Symptoms” followed three phases (Figure 3): phase 1, identification of needs [6]; phase 2, design and development; and phase 3, feasibility study. This paper reports on the usability studies conducted in phase 2 (Figure 3). The results from this study informed the content and structure of the program used in the feasibility study.

The objectives of the present study were (1) to investigate the usability of the “My Symptoms” program with a specific focus on how to improve functionality and navigation and (2) to explore how users could help improve the intuitiveness and user-friendliness of the program.

## Methods

### Overview

From 2020 to 2021, the last part of the development phase 2 (Figure 3) commenced with an emphasis on the usability of the program. To examine user experience with navigation and functionality in the program, we conducted thinking-aloud sessions asking participants to speak aloud while completing various tasks [13,14].

The project group developing the self-help program consisted of HCPs (GPs, a psychiatrist, psychologists, and a public health scientist), anthropologists, techno-anthropologists, and web developers. The techno-anthropologists conducted the thinking-aloud sessions, whereas the whole group was involved in the processing of results.

### Ethical Considerations

All participants were informed orally and in writing about the study, and all participants gave their consent to participate. The study was approved by the Danish Data Protection Agency (J. no. 1-16-02-16-19). The Danish Act on Research Ethics Review of Health Research Projects is not applicable to qualitative studies. Therefore, ethical approval was not required from the Committee on Health Research Ethics in the Central Denmark Region.

### Participants

We included a convenience sample of primary care patients and laypeople. In all, 6 GPs identified and invited 4 patients aged 18-65 years with PPS. These potentially eligible patients were informed orally and in writing about the project by the GPs and gave their consent to be contacted by a researcher from the project team. A project member screened consenting patients according to the selection criteria and finally included or excluded patients (Textbox 1).

**Selection criteria for patients.**

**Inclusion criteria**

1. Age 18-65 years
2. Affected by persistent physical symptoms according to their general practitioner
3. “Somewhat bothered” by at least 4 of 25 symptoms (scoring ≥2 on a Likert scale with each symptom from 0 “Not bothered at all” to 4 “Bothered a lot” by the Bodily distress syndrome (BDS) checklist [15])
4. Speak and understand Danish

**Exclusion criteria**

1. Severe mental disorder
2. Sick leave for more than 8 consecutive weeks

We aimed for the inclusion of approximately 12 patients, considering the number of 5-8 participants proposed by Nielsen [16] and Virzi [17] on finding most of the usability issues. Moreover, when investigating usability iteratively, the number of participants needed is adjusted continuously based on data saturation, that is, in our case, the number of medium and critical issues. However, the restrictions caused by the COVID-19 pandemic [18] challenged the recruitment process in general practice. To finalize the study within its time limits, we therefore chose to supplement the user inclusion with 7 laypeople recruited through personal networks. As bothersome symptoms are a general phenomenon [19], we expected laypeople to be able to relate to current or prior symptom experiences. The patient group consisted of 4 patients; 50% (n=2) were female, and ages ranged from 24-57 years. One patient had 2 rounds of testing and interviewing, whereas the remaining patients had 1. The laypeople group consisted of 7 individuals; 57% (n=4) were female, and ages ranged from 20-31 years. In all, 3 laypersons had 3 rounds of testing and interviewing, 2 had 2 rounds, and 2 had 1 round.

### Usability Investigation by Thinking Aloud

To investigate usability, we applied the thinking-aloud method. The aim of this method was to “capture” the users’ thoughts as they navigated the “My Symptoms” program to gain insight into how they experienced the program in the context of actual use and what they found easy or difficult to do or understand [20]. The project group translated these verbalized thoughts into specific changes that needed to be made in the program.

Due to the restrictions caused by the COVID-19 pandemic, we conducted the thinking-aloud sessions on the web [18]. We used screen sharing that allowed for easy observation of how the user navigated the program and recorded the sessions for subsequent analysis. We chose to let the users’ symptoms guide their way through the program to approximate actual use case scenarios [21]. When investigating the functionality of the interactive exercises, predefined tasks were provided with scenarios based on interviews from a preceding study [6]. The users were told to imagine being referred by their GP, coming home with a flyer with instructions on how to access and use the program. In the first of 3 rounds of testing, all users carried out the same 8 tasks related to log-in, filling out questionnaires and exercises, finding information about one’s symptom(s), and using interactive behavior change tools. For example, the interviewer would ask the user to access information about the most bothersome symptom and observe their behavior. Sometimes when issues arose, the interviewer asked, “what did you expect would have happened?” but mostly kept quiet until the participant had completed a task to not interfere with the participant’s experience.

Rounds 2 and 3 focused more on testing predefined, specific elements in the program rather than core functionality. From the first round of testing, we observed that laypeople and patients interacted similarly to buttons, sliders, and other interactive web elements, which was why we included more laypeople than patients for these rounds. Immediately after thinking aloud, a follow-up interview [22,23] was conducted inquiring about the users’ experience of the program. The questions were related to overall experience, relevance for everyday life, and the use of the internet for behavior change. The thinking-aloud testing and follow-up interview lasted 45-90 minutes. A total of 20 sessions were conducted.

### Data Analysis

All audio and video recordings were transcribed. Based on transcripts and notes taken during the thinking-aloud sessions, we identified usability issues and rated the severity of these as minor, medium, or critical. Encountered bugs were also flagged. The GitHub platform (GitHub, Inc) [24] was used to relay and manage bugs and usability issues to the web developers.

Using a thematic analysis approach [25], all usability issues rated as medium or critical were coded based on content. Subsequently, these codes were mapped to 2 predefined categories: navigation and functionality. After categorization, we cross-referenced the emerged categories with general usability heuristics [26]. The usability heuristics offer a set of guidelines curated over decades of designing systems and identifying usability problems. They are often used to inform design decisions by experts within the field of human-computer interaction.

## Results

### Categories and Core Issues

The thinking-aloud sessions gave insight into functionality and navigation. Additionally, the sessions revealed potentially problematic phrases and wordings that hindered the usability of the program. Inductively, a third category concerning content emerged, leaving us with 3 overall categories of issues. In Table 1, the results are presented according to these categories: functionality, navigation, and content, using examples from the data and the issues identified. In the following sections, we elaborate on some of the issues found within each category. These issues are marked by a footnote.

**Table 1:** Overview of results with categories and core issues.

Categories	Core issues
Translating therapy into digital functionality	Missing information on how to perform exercises^a^ Interactive exercises often need surrounding text before being used sufficiently Using examples of changing habits as options and not suggestions Confusion about “Archive” and “Close” buttons when using core tools
Designing navigation	Too many submodules and pages^a^ Missing references for getting back to already known content Missing navigation buttons at the bottom of pages^a^ Too much blank space leaving users to miss content
Content guides program use	Text-heavy pages^a^ Using quotation marks on health information decreases users’ perceptions of program legitimacy^a^ Missing language directed at the user

^a^These core issues are further elaborated below.

### Translating Therapy Into Digital Functionality

In the development phase, the interactive elements and exercises of the “My Symptoms” program required close collaboration between web developers and the project group as common therapeutic tools were translated into digital equivalents. The usability test revealed that the intended purpose of the therapeutic content in some instances had been lost in this translation. General examples of core issues that suffered from translational issues were missing information on how to perform exercises, the need for additional text or content to explain exercise use, generic examples on changing habits were not translated by the user and used as is, and confusion on how to use core tools such as the “Goal Staircase.” Most of these issues concerned exercises that had been translated from a physical context into digital entities. Such translational problems were especially evident with the “Sorting of Values” [27] exercise.

**Figure 4:**
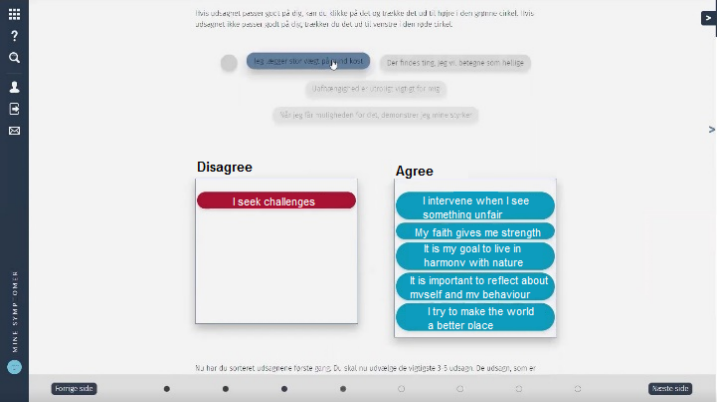
The "Sorting of Values" exercise in the "My Symptoms" program. Statements such as "I seek challenges" and "My faith gives me strength" must be sorted in columns of "disagree" (left) or "agree" (right).

In the “Sorting of Values” exercise, the user was supposed to select statements about the life values most important to him or her, such as “I value a healthy diet,” “I look for challenges,” and “My goal is to live in harmony with nature.” The exercise was then to sort the statements into the columns of “agree” or “disagree.” Finally, the user was prompted to choose 3 to 5 of the agreed statements into a new column to identify the most important values. Figure 4 shows how the web developers and the HCPs had manifested the concept of the exercise.

**Figure 5:**
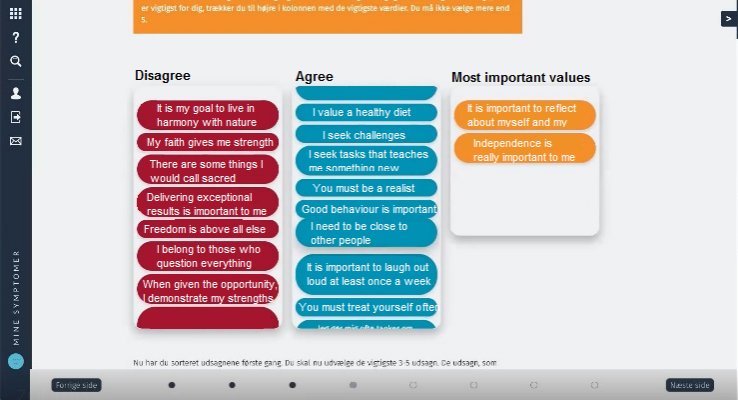
The "Sorting of Values" exercise showing the third column, "the most important values" (yellow), used to store and remember statements for later use in the module.

In the “Sorting of Values” exercise, only 1 of 7 users was able to complete the exercise. Completing this exercise was critical since the rest of the module depended on the “results” gained by completing it. This 1 user was able to access a “hidden” column that would only appear when *all* the value-statements had been sorted into the 2 above columns. Only then the user was able to sort the 3 to 5 most important statements into the previously hidden column, and the program would store these for later use. Figure 5 shows what the exercise looks like when completed.

In the physical version of the exercise, the patients would hold a deck of cards in their hands that would give them tactile feedback on how many cards or statements were left to sort. With the example of the “Sorting of Values” exercise, we noticed that one reason for stopping the sorting of values into the first 2 columns was the absence of knowledge on how many statements have to be sorted and how long it would take. Thus, the users figured it would be okay having some of the statements sorted into the 2 columns and moved on to the next page. The heuristic “visibility of system status” [26] reminds us that the user in general should be kept informed on the state of the program, for example, through feedback on the current progress of a specific exercise or task.

**Figure 6:**
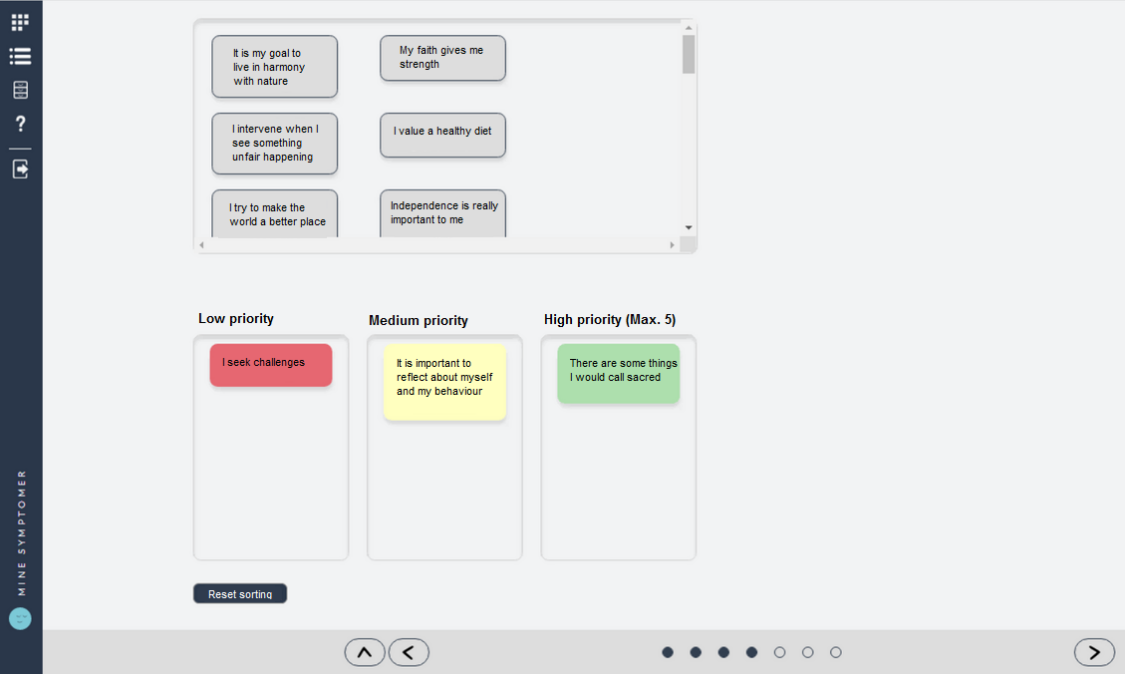
The redesign of the "Sorting of Values" exercise showing the third column at all times, the added "scroll box," and the reset button.

Based on the findings, the project group developed a solution where the third column was now shown all the time (Figure 6) to display the goal of the exercise from the beginning. Furthermore, statements were grouped into a “scroll box,” hinting at the number of statements and how many needs to be sorted. Additionally, a reset button was added to allow for increased user control.

The “Sorting of Values” exercise was one example of several issues on not getting enough information at the right time to complete the exercises. Other issues were, for example, related to missing tactile information such as the “Sorting of Values” exercise and missing guidance from a therapist in the digital format.

### Designing Navigation

When investigating navigation, core issues were regarding too many pages and submodules, the placement of navigation buttons, navigating back to known content, and too much blank space. These navigational issues were especially frustrating to new users trying to find their way through the program.

An example of a major navigational issue identified was navigating the different levels of the program. Knowing what page the user was on in relation to the rest of the program proved difficult. This was important if the user wanted to repeat or go back to an exercise in a new sitting. The thinking-aloud sessions demonstrated how most people tried to remember the location of the exercises by recalling the modules and the title on subpages. As a module could hold up to 3 levels and 10 subpages, this strategy proved difficult. Furthermore, the subpages of the modules were navigated by the next and previous page buttons bound to the top of specific pages. This made the navigation buttons disappear when scrolling down.

To alleviate the users’ need for recalling the different locations of the exercises, a “sticky” navigation bar was developed. This was done in reference to, for example, the heuristics “recognition rather than recall” and “visibility of the system status” [26]. The new navigation bar indicated at which level and page the user was, and it was shown all the time at the bottom of the page. We also reduced the number of levels of all modules to 2. Moreover, a breadcrumb trail was made visible showing the full extent of the path.

Initially, the program was aimed at allowing the users to navigate freely according to their symptoms and to let them decide what content would be appropriate or helpful to explore. However, the usability tests revealed a need to help the users better navigate between the overall modules. They needed a better framework for their journey in the program. Therefore, we decided to let the first 3 modules open gradually before providing free access to all modules. When a new module opens, the users are prompted with a text message on their telephone.

### Content Guides Program Use

Analysis of the thinking-aloud and interview data revealed that specific program content could potentially hinder adequate program use. Core issues in this category were related to pages being text heavy, the perception of program legitimacy, and the use of language directed at the user. These issues could in many instances be attributed to the lack of therapeutic assistance to mediate program use, thus compensating for it with more text and content. These issues were especially evident when comparing feedback from patients and laypeople. Patients were more focused on the framing of the content in the program than laypeople. For example, in content, quotation marks were used to stress some of the medical terms. Most patients responded poorly toward this usage and figured the terms were made up, making them question the legitimacy of the program and their own experience with their symptoms. This was not an issue with laypeople. Patients also spent more time investigating content as means of navigation and interaction, even though we explicitly stated that reading the content thoroughly was not necessary during the usability testing. Contrarily to patients, laypeople found it bothersome to read too much text as a guide for navigation and interaction. Instead, they preferred being guided by different design cues. Patients, however, found it necessary to read most of the text to make sure they were on the right path while being guided by design cues. Patients who experienced symptoms fatigue and lack of concentration were unable to adhere to the thinking-aloud tasks to the same degree as other users, which they attributed to the pages being too text heavy. With patients tending to read most texts thoroughly, the program may be experienced as being too bothersome, thus decreasing motivation for use.

In the follow-up interviews, we found that users related to the content in different ways. For example, when we asked participants, “Are there elements in the program you would like to return to and if so which ones?” (Table 2), laypeople spoke more in terms of *what* elements they would use, and patients spoke more in terms of *how* they would use them.

**Table 2:** Excerpts from the follow-up interviews (translated from Danish).

Elements	Laypeople	Patients
My feelings	“I think it is interesting to see how my feelings can affect my physical symptoms and vice versa. That is useful also in a general sense” (Layperson 5)	“I like the connections between my feelings and symptoms, but they require a lot of energy to make. I would need to find a calm space, like the woods, where I can be with my thoughts without too many distractions. Then it would just be myself and my iPad” (Patient 2)
My sleep	“Yeah, that with the sleep patterns, I think I could use. The sleep registration tool” (Layperson 7)	“I haven't really tried those things before. It seems like a good idea, if you want to gradually go earlier to bed; go to bed one hour earlier every day using the Goal Staircase. But, I’d might just use some paper to keep track of my sleep” (Patient 3)

## Discussion

### Principal Findings

The purpose of this study was to investigate the usability of the “My Symptoms” program by exploring functionality and navigation from a user perspective. Investigating the functionality of the program mostly revealed issues related to the translation of face-to-face therapeutic material into digital exercises. This was especially in relation to issues regarding the lack of transparency on how to complete exercises and lack of continuous feedback on exercise progression. With regard to navigation, most of the usability issues were about the number of subpage levels and lack of markers when users wished to go back to exercises or content. By reducing the number of levels of subpages and using different design cues, we sought to evoke the recognition of symbols rather than recall of page titles.

The content of the program was not the target of our investigation but became a stepping stone for understanding how content also guides navigation and functionality. This was especially evident from the patients’ feedback. Although, differences in the inclusion of patients and laypeople were found, including laypeople was helpful when there was a lack of patients or patients were challenged cognitively—especially since the experience of bothersome physical symptoms is a common phenomenon. Laypeople and patients draw on the same type of IT-related schemata, for example, knowledge of browsing the web, using social media, and more. Moreover, they also draw on the experience on common symptoms.

### Discussion of Results

Designing behavior change exercises requires a lot of attention to the communication between the HCPs and web developers. The process of improving the functionality of the “Sorting of Values” exercise was attributed to the iterative process of continuously testing the exercise with users. Testing the exercise multiple times helped us make a digital therapy that makes sense at all levels: therapeutic model, technical capacity, and user-friendliness. Not testing the usability would have made the exercise, and the rest of the “My Values” module, inaccessible to most users. Here, a pragmatic approach of understanding both the therapeutic models and the system capability was necessary. For example, the iterative process allowed different versions of the same exercise to be investigated multiple times by different participants, making the users, web developers, and HCPs all a part of the designing process [12,28]. The user feedback also helped inform uncertainties within the project team on deciding specific design solutions. Furthermore, a systematic review on user involvement in the development of patient decision aids found that projects could be more iterative and that reporting on the differences in design changes between iterations could help explain the rationale behind the finale product [29]. Here, we used the “Sorting of Values” exercise as an example of what rationales went into developing it and how we came about it.

In this study, laypeople acted as surrogates for patients. Knowing when and how to include laypeople is valuable when there is a lack of the intended end user. The use of surrogates can be beneficial in getting rid of the most critical usability issues before gaining access to a limited end user group [30]. When using surrogates, one must consider to which degree the real end user and the surrogate share the same characteristics [31]. In this study, the focus on common symptoms created a general foundation for testing the program for both patients and laypeople. By creating task scenarios based upon the real end users’ needs found in a preceding study [6] and comparing the real end users who are available to the surrogates, we were able to come up with and iteratively tailor the usability investigation in favor of our real end users. Using laypeople and patients in testing the usability of the “My Symptoms” program thus offered great insights to the general navigation and functionality. With patients experiencing, for example, frequent headaches, some lower back pain, and others fatigue, there was a widespread variation of how the patients’ symptoms manifested into in-program behaviors.

### Strengths and Limitations

Through the thinking-aloud tests, we observed user behavior, and through the interviews, we obtained comments and suggestions for design changes. These data were cross-referenced with Nielsen’s heuristics [26]. Using the heuristics in combination with user-generated data, we wanted to alleviate the concern of bias from letting design changes being solely driven by experts’ interpretation of the heuristics [32], which we consider to be a strength.

Although the web-based tests and interviews made for easy observation and recording, it limited our contextual understanding of the practical setup and the environment of the users in which they might use the program. Likewise, our aim for consistency in testing solely with a desktop setup did not necessarily match our patients’ use cases, pointing toward further investigations into the use of, for example, tablets and smartphones, when using the “My Symptoms” program. Thus, future research on improving the “My Symptoms” program and other studies alike could benefit from focusing on contextual inquires [33] with patients both via observations and self-reported data that are, for example, enabled by different kinds of cultural probes [34].

In usability engineering, it is well known that by understanding the user’s mental model, or schemata, we are able to come closer to a conceptual model [35] by which we can come up with suggestions for changing the program. Moreover, the interviews with patients and observations in general practice preceding this study [6] helped inform the patient use cases when making the task scenarios for the thinking-aloud investigation. This process was similar to the concept of creating personas [36,37]. This also helped us make more realistic scenarios for including laypeople. However, even though laypeople were able to provide useful knowledge on their experience in the program—navigating out from their mental model [35] in a way similar to patients—laypeople cannot provide us with the experience of testing the program as someone experiencing persistent symptoms. As the patients pointed out, pages may be too text heavy; thus, a greater focus on health literacy is warranted. Therefore, although bodily sensations and symptoms are part of human life, recall bias on part of laypeople may exist, pointing to a limitation of this study.

Although the small sample size could be seen as a limiting factor, using the thinking-aloud method to highlight usability problems of immediate use is known to be an effective tool when having a small sample size. Because of the amount of detailed data the method provides, only 5-8 users are necessary to detect 80% to 85% of the usability problems [16,17]. Furthermore, according to a scoping review [38], different open-ended qualitative investigations should be deployed instead of a single method [39-41]. Second, immediate use should be explored rather than only retrospective investigations, such as interviews [42]. Third, during the development stage, it may be more beneficial to be informed by in-depth investigations using multiple cycles of exploration [28] and tests with a smaller group of participants than using a larger group of participants only once [38].

Interviewing the users before and after the thinking-aloud test enabled us to understand to which degree the users were accustomed to using web applications and IT in general. The interviews also gave us a chance to know how the patients were bothered by symptoms, helping us understand how the symptoms might affect the patients’ in-program behavior. Furthermore, the interviews helped us dive into issues that occurred during the thinking-aloud test, giving the users a way to suggest design changes retrospectively.

### Conclusions

Creating a digital self-help treatment program demands special attention to user-friendliness and intuitiveness. Program usability can make the difference between getting the needed treatment or not. User feedback helped improve the usability of the program and revealed how therapy sometimes was lost in the digital translation. This was especially relevant in relation to the themes: functionality, navigation, and content. Here, reducing the number of subpages, providing users feedback on tasks, and being sensitive to the framing of the content increased user satisfaction. Using the thinking-aloud method and heuristics in combination enabled us to upscale the specific design iterations into more broadly defined design statements that are applicable throughout the program. Furthermore, the usability testing helped facilitate knowledge sharing between different professions as a precondition for a successful program development facilitated by an iterative development process.

## Abbreviations

GP: general practitioner
HCP: health care professional
PPS: persistent physical symptoms
